# Electric field-induced crystallization of ferroelectric hafnium zirconium oxide

**DOI:** 10.1038/s41598-021-01724-2

**Published:** 2021-11-15

**Authors:** Maximilian Lederer, Sukhrob Abdulazhanov, Ricardo Olivo, David Lehninger, Thomas Kämpfe, Konrad Seidel, Lukas M. Eng

**Affiliations:** 1 Fraunhofer IPMS, Center Nanoelectronic Technologies, 01109 Dresden, Germany; 2 Institut für Angewandte Physik, Technische Universität Dresden, 01062 Dresden, Germany; 3 Center of Excellence—Complexity and Topology in Quantum Matter (ct.qmat), Technische Universität Dresden, 01062 Dresden, Germany

**Keywords:** Electronic devices, Ferroelectrics and multiferroics, Phase transitions and critical phenomena

## Abstract

Ferroelectricity in crystalline hafnium oxide thin films is strongly investigated for the application in non-volatile memories, sensors and other applications. Especially for back-end-of-line (BEoL) integration the decrease of crystallization temperature is of major importance. However, an alternative method for inducing ferroelectricity in amorphous or semi-crystalline hafnium zirconium oxide films is presented here, using the newly discovered effect of electric field-induced crystallization in hafnium oxide films. When applying this method, an outstanding remanent polarization value of 2P$_{\mathrm{R}}$ = 47 $\upmu$C/cm$^{2}$ is achieved for a 5 nm thin film. Besides the influence of Zr content on the film crystallinity, the reliability of films crystallized with this effect is explored, highlighting the controlled crystallization, excellent endurance and long-term retention.

With the discovery of ferroelectric hafnium oxide^1^ its application in many novel device concepts like non-volatile ferroelectric FETs^2^, ferroelectric tunnel junctions^3^, as well as pyro- and piezoelectric sensors^4–6^ has been explored. This is a direct consequence of hafnium oxides full complementary–metal–oxide–semiconductor (CMOS) compatibility and its outstanding properties, like high coercive field and scalability to ultra-thin films^7^. As many HfO$_{2}$-based devices can be integrated into the back-end-of-line (BEoL), recent publications focused on hafnium zirconium oxide (HZO), which enables crystallization at low temperatures, compatible with the BEoL thermal budget requirements^8,9^.

However, recent works discovered a new effect in the hafnium oxide system, namely electric field-induced crystallization^10^. This allows to apply electric fields in order to crystallize the hafnium oxide film directly into the ferroelectric phase, as illustrated in Fig. 1a, with an effective activation energy of 0.45 eV^10^. Nevertheless, this effect is still not understood in detail. Therefore, the behavior of this effect for hafnium zirconium oxide films with different Zr content as well as the reliability of those films is investigated here. Samples with initially high amorphous phase fractions showed excellent endurance and no detectable retention loss. Furthermore, the degree of crystallization and in consequence polarization can be tuned in a very controlled manner, allowing common ferroelectric operation with a selected maximum remantent polarization (P$_{\mathrm{R}}$).

For investigating electric field-induced crystallization in HZO films, 5 nm thick HZO films with a Hf:Zr cycling ratio of 3:1, 5:3, 1:1, 3:5, and 1:3, respectively, were grown. More details on the material and device fabrication can be found in the Methods section. All films received a rapid thermal processing (RTP) spike anneal at 450 $^\circ$C for 60 s to form nuclei required for electric field-induced crystallization^10^. Grazing-incident X-ray diffraction (GIXRD) patterns (see Supplementary Fig. S1) reveal X-ray amorphous films for a high Hf content. Films with higher Zr content are semi- or fully-crystalline.

**Figure 1:**
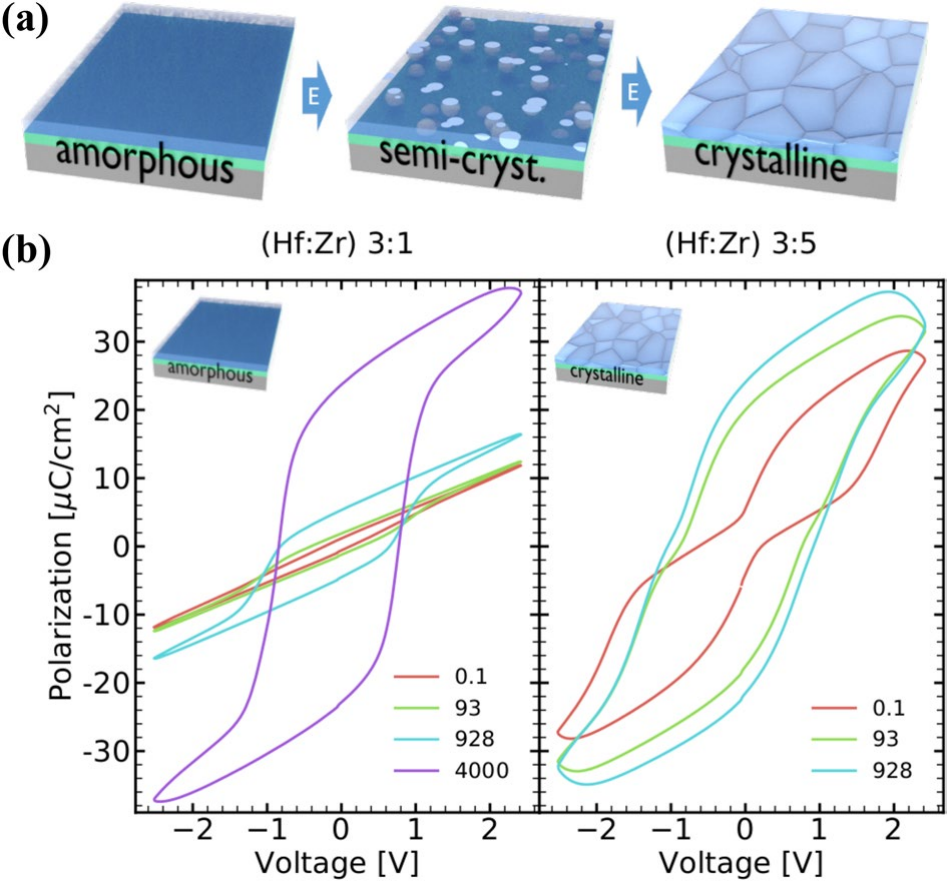
Electric field cycling of hafnium zirconium oxide can result in a field-induced crystallization, as shown schematically in (**a**). Pristine degree of crystallinity, based on GIXRD results, is given alongside. The evolution of the polarization hysteresis of a Hf- and Zr-rich sample (**b**) upon electric field cycling. Dielectric behavior is observed initially for the amorphous Hf-rich sample. The crystalline Zr-rich sample behaves antiferroelectric-like initially.

**Figure 2:**
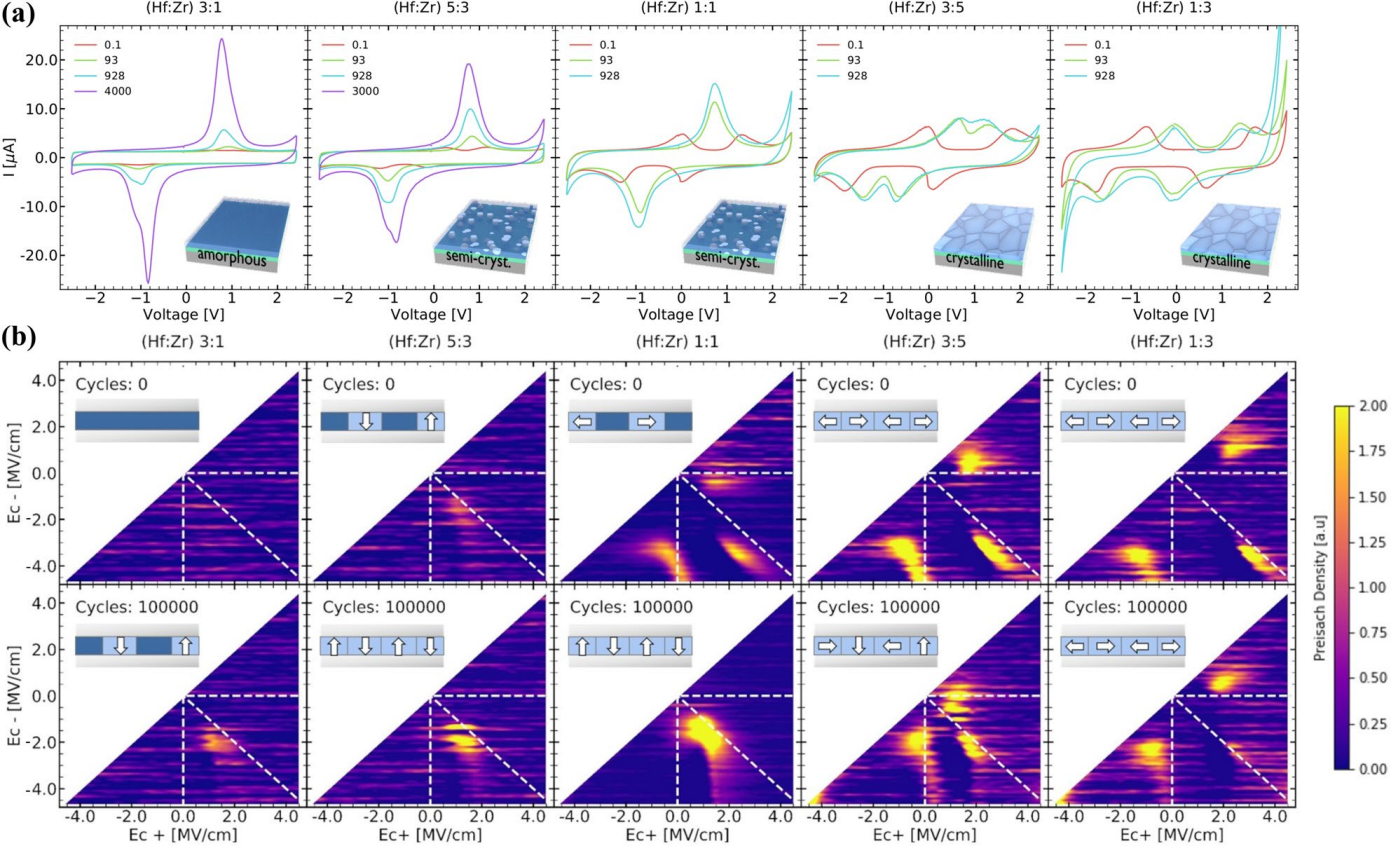
Changes in the displacement current response (**a**) with electric field cycling at 2.3 V for samples with different Zr-content. Hf-rich samples are X-ray amorphous or semi-crystalline and show a increase in the displacement current peak amplitude, whereas Zr-rich samples are (semi-)crystalline and antiferroelectric-like wake-up. First-order-reversal-curve (FORC) measurements (**b**) show peak changes in the intensity and peak splitting for the Preisach density, which can be related to electric field-induced crystallization and $90^\circ$-domain wall motion (ferroelastic switching), respectively.

The polarization response (see Fig. 1) of these samples behaves quite different for high Hf or Zr content, respectively. While the Hf-rich sample shows sudden wake-up^10^ described by a transition from dielectric to ferroelectric behavior, the Zr-rich sample shows classical wake-up, starting antiferroelectric-like (AFE-like) and becoming ferroelectric-like upon electric field cycling. Moreover, outstanding 2P$_{\mathrm{R}}$ values of 47 $\upmu$C/cm$^{2}$ are observed for the Hf-rich sample. This is quite remarkable since these values exceed even the crystalline Zr-rich film. As crystallinity, or rather a crystalline phase with a non-centrosymmetric space group, is required for the presence of ferroelectricity, this clearly indicates electric field-induced crystallization for the Hf-rich films.

To compare the samples in more detail and to assess the degree of sudden and classical wake-up contributions, the displacement current is analyzed (see Fig. 2a). For sudden wake-up, as strongly present for Hf:Zr 3:1, an increase in the integrated switching peak intensity is clearly observable. Consequently, this process can be clearly separated from filament formation in HfO$_{2}$-based resistive random access memory devices, which is described by a leakage current increase. On the other hand, classical wake-up^10^ (Hf:Zr 1:3) shows initially two split switching peaks in each direction. Upon cycling these two merge closer, while the total integrated switching peak intensity stays approximately constant.

For intermediate Zr concentrations, a superposition of both behaviors can be observed. Two very faint switching peaks, close to each other are observed for Hf:Zr 5:3. However, a single merged peak is observed even after low amounts of cycles, increasing continuously in intensity with further cycling. For the Hf:Zr 1:1 sample, the initial presence of classical wake-up is clearly apparent. Nevertheless, an increase of the integrated intensity is still observable with cycling. This nicely fits to the aforementioned GIXRD pattern, where this sample appears semi-crystalline. For even higher Zr content (Hf:Zr 3:5), no significant contributions of sudden wake-up are observable, in-line with the crystalline GIXRD pattern.

In addition to the change in wake-up behavior with increasing Zr content, an increase of leakage current is observed as well. This indicates that a higher Zr content may become detrimental for device applications. However, this is in comparison with amorphous or semi-crystalline Hf-rich HZO films, which do not contain larger amounts of monoclinic phase, which would be present in thermally crystallized Hf-rich HZO films. These have been reported to exhibit earlier breakdown compared to Zr-rich AFE-like films^11^. Furthermore, it confirms that oxygen vacancy migration and consequently filament formation is not interweaved with the crystallization process.

In order to gain deeper insight into the physical mechanisms in these samples, first-order reversal curve (FORC) measurements were conducted and the switching (Preisach) density was extracted (see Fig. 2b). More details on this method can be found in literature^12^. For increased Zr content, clear peak splitting into three distinct peaks is visible. However, a continuous shift of these peaks is observed with decreasing Zr content and increased cycling, moving from a pinched behavior to an AFE-like behavior, as indicated by the horizontal and vertical dashed lines. While this cannot be easily explained by a tetragonal-orthorhombic phase transition^13^, such behavior can be related to ferroelastic-mediated switching, which was recently demonstrated to be the physical mechanism behind AFE-like behavior in HfO$_{2}$ material system^10^. The previously presented temperature and frequency dependence of the AFE-like wake-up, contradicts diffusion processes and domain wall pinning^10^. In addition, transmission Kikuchi diffraction did not observe a significant monoclinic phase fraction prior wake-up and $90^\circ$ re-orientation of the polarization axis to the out-of-plane orientation post cycling^10^. Combined with the high Curie temperature $(>250^\circ {\rm C})$^14–18^ and displacement measurements^5^, transitions from non-polar to polar phases are not supported by the experimental results as an origin for AFE-like behavior. However, antipolar orthorhombic phases could still be present alongside ferroelastic-switching^10^.

In consequence, the increase of AFE-like behavior with higher Zr-content can be related to an increased tensile stress and therefore an in-plane domain formation combined with $90^\circ$-domain wall motion (ferroelastic switching). This is most likely related to the increased crystallinity in these layers, which enables the promotion of mechanical stress. The increased crystallinity for higher Zr-content is a direct result of the reduction in crystallization temperature.

For Hf-rich layers, the crystallization occurs during cycling, as confirmed by transmission electron microscopy (TEM) analysis of a cycled device (see Fig. 3). As mentioned previously, these layers behave ferroelectric due to the presence of a displacement current peak. This can be observed in the Preisach density as well, increasing in intensity upon cycling. Consequently, no significant amounts of ferroelastic-switching should be present in these films. Therefore, an out-of-plane domain structure and $180^\circ$-domain wall movement are expected, as indicated by Fig. 2b. In the case of minor AFE-like behavior (slightly pinched hysteresis), both switching processes and domain configurations can be present.

In order to understand, how good the crystallization in these samples can be controlled, films are cycled up to a defined number of cycles with an amplitude of 2.3 V. Then the stability of this state is checked by cycling at 1.9 V (see Fig. 4). As seen here, the crystallization is stopped at lower voltage amplitudes, and a common ferroelectric behavior without cycling variation in P$_{\mathrm{R}}$ can be observed. In consequence, the maximum polarization and crystallinity of the film can be controlled very accurately, allowing for realizing new device concepts based on this effect.

**Figure 3:**
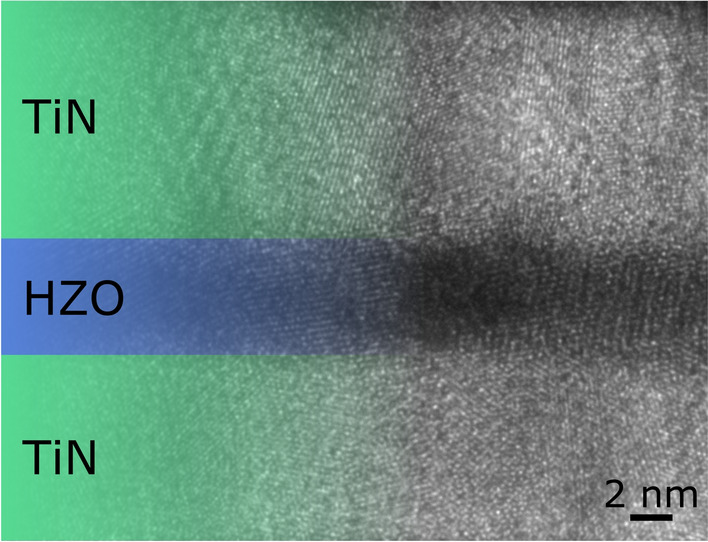
Transmission electron microscopy image of a cycled device with a 5 nm HZO layer, which behaved X-ray amorphous initially.

**Figure 4:**
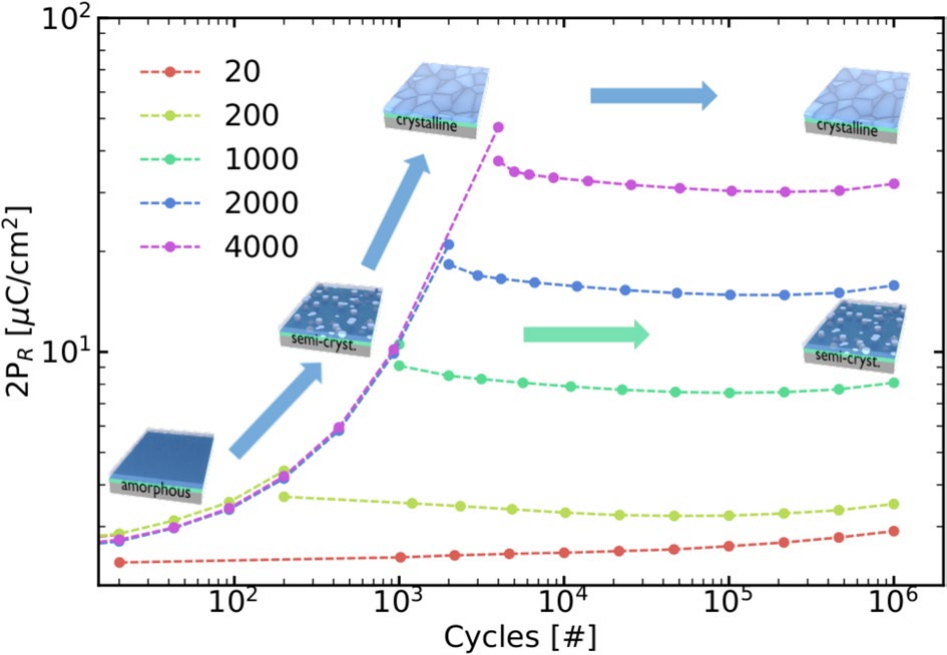
Electric field cycling at 2.3 V up to a certain amount of cycles and subsequent cycling at 1.9 V allows for operating the HZO layer at different degrees of crystallinity reliably up to 10$^6$ cycles. No significant change in remanent polarization and in consequence crystallinity is observed for the lower cycling amplitude.

**Figure 5:**
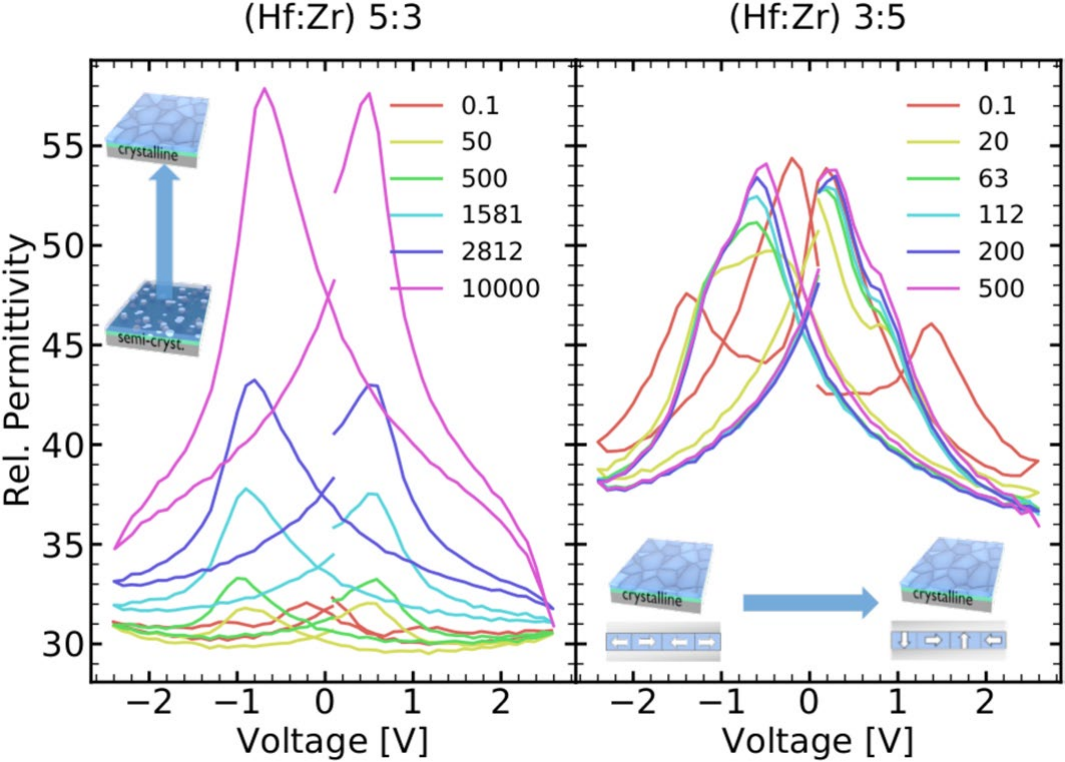
Capacitance measurements after different amounts of electric field cycles show a strong increase in permittivity as well as the ferroelectric “butterfly” effect for the Hf-rich sample, in agreement with field-induced crystallization. For the Zr-rich sample, no significant change in permittivity, but changes in the (anti-)ferroelectric response are observed, which are likely originating from ferroelastic-mediated wake-up.

This effect can be observed as well in the capacitance and permittivity of the sample (see Fig. 5). Here an increase of permittivity appears with electric field cycling for Hf-rich samples, due to the crystallization into the ferroelectric phase, whereas only minor changes are observed for the crystalline Zr-rich films, due to the reorientation of the polarization axis. This behavior is very interesting for the implementation of HZO as a ferroelectric thin film varactor^19–21^. The Hf-rich samples show a high capacitance tunability, as demonstrated in Fig. 5, exceeding the tunability of Zr-rich sample.

Finally, the reliability of these films is investigated. No significant retention loss is observed for the ferroelectric films (see Supplementary Fig. S2). Only the film Hf:Zr 1:3, which behaves AFE-like, shows retention losses, which is an artifact of the imprint effect in this measurement, due to the initially low P$_{\mathrm{R}}$ value.

For endurance, the films are field-cycled at 2.3 V for 3000 cycles, to wake-up/crystallize the films. Afterwards, the films are cycled at 1.9 V for $10^{8}$ cycles. As observed here (see Supplementary Figs. S3 and S4), the Zr-rich film breaks down already during the wake-up cycling. The other two layers however survive the wake-up field stress. Moreover, the difference in the wake-up mechanism clearly appears in the P$_{\mathrm{S}}$ evolution. Here, the initial crystallinity is reflected by the initial P$_{\mathrm{S}}$ value, which does not increase significantly for fully crystalline films. For sudden wake-up, a strong increase of P$_{\mathrm{S}}$ is observable, finally reaching values like the fully crystalline film.

As these films survived the wake-up, no significant degradation is observable until $10^{7}$ cycles. Afterwards, a soft breakdown is observed via increased leakage current. As the shape of this breakdown is identical for the X-ray amorphous and semi-crystalline sample, no reduction of endurance is expected for films crystallized by sudden wake-up.

**Figure 6:**
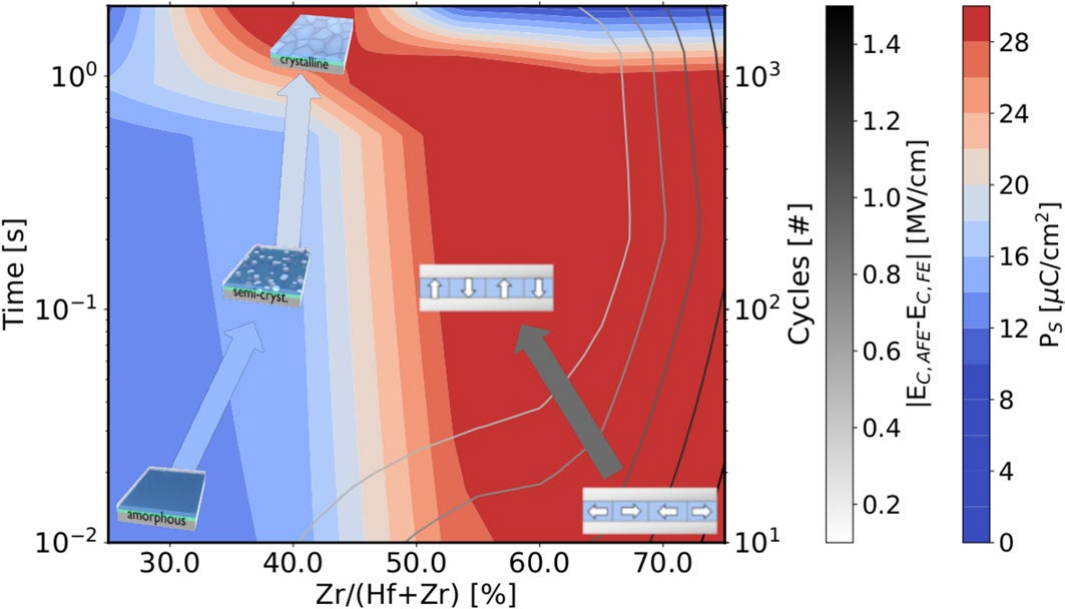
Visualizing P$_S$ and the the peak splitting distance over cycling time/cycle number, an amorphous-crystalline phase boundary as well as the in-plane to out-of-plane transition of the ferroelectric axis appear, respectively. Since the field-induced crystallization correlates with time and the AFE-like wake-up with the cycle number, those two transitions can be moved in opposite directions depending on the cycling frequency.

Based on the previous analysis, the field cycling data allows to construct a cycling-dependent phase diagram (see Fig. 6). Here, P$_{\mathrm{S}}$ can be used to describe the crystallinity of the sample, as discussed above. The reduction of P$_{\mathrm{S}}$ for high cycle numbers in Zr-rich samples is due to breakdown. Moreover, the degree of AFE-like behavior, here approximated by the displacement current peak distance to the ideal ferroelectric coercive field value ($|E_{C,AFE}-E_{C,FE}|$), can be summarized as well. A quite sharp transition from amorphous to crystalline can be observed. This amorphous-crystalline phase boundary is clearly concentration and cycling time dependent. The latter follows an Arrhenius behavior, as confirmed previously^10^. The transition from AFE-like behavior, resulting from in-plane polarization orientation, to out-of-plane domains (ferroelectric behavior) is quite smooth. In addition, it has been shown that this process is dependent on the cycling number^10^. As the relationship between cycling time and number is described by the cycling frequency, this allows for tuning these two effects to achieve a desired cycling behavior.

In conclusion, electric field-induced crystallization of ferroelectric HZO has been demonstrated to be a very controlled and reliable effect. While the initial degree of crystallization and antiferroelectric-like behavior can be controlled via the Zr-content, the crystallization of semi-crystalline and X-ray amorphous films can be controlled very accurately. Moreover, regular ferroelectric switching can be performed at a selected degree of crystallinity without changing the maximum remanent polarization. No significant retention losses and an endurance of more than 10$^{7}$ cycles at stable remanent polarization values were observed. Finally, a cycling and concentration dependent phase diagram can be constructed that provides guidelines on how to tune AFE-like wake-up and field-induced crystallization separately.

## Methods

### Material preparation

The test structures were fabricated in metal-ferroelectric-metal (MFM) capacitor configuration, where HZO thin film was capped between titanium nitride (TiN) thin film electrodes. The structures were deposited on top of highly doped 300 mm silicon wafers, that acted as a bottom ground for electrical measurements. A 10 nm bottom TiN electrode was deposited with atomic layer deposition (ALD), utilizing the TiCl$_{4}$ and NH$_{3}$ precursors and the 10 nm top TiN electrode was deposited by reactive magnetron sputtering. The HZO layer was deposited by ALD with HfCl$_4$ and ZrCl$_4$ precursors, and H$_2$O as the oxidizing reactant. The Hf and Zr precursor cycling ratio was varied between 3:1, 5:3, 1:1, 3:5, and 1:3. The thickness of the films was set to 5 nm. The samples were annealed by a rapid thermal anneal (RTA) at 450 $^\circ$C for 60 s in N$_{2}$ atmosphere. For the further electrical measurements the Ni/Pt dot contacts were sputtered through a shadow mask on top of the MFM layers by using an electron beam evaporation. The dots were later used as a hard mask for a wet-chemical etching of the top TiN layer with SC1 solution (NH$_4$OH + H$_2$O$_2$) and thereby forming capacitor’s active area.

### Electrical characterization

The electrical measurements were carried out with Aixacct TF 3000 analyzer. For the field-induced crystallization and wake-up a square waveform of 20 Hz frequency with an amplitude of 2.3 V was used. Such a low frequency was chosen to better observe the development of electrical properties between cycles. The maximum number of crystallization/wake-up cycles was varied between 100 and 4000. For the endurance cycling, the amplitude was reduced to 1.9 V to avoid the breakdown due to high leakage currents. The maximum number of endurance cycles was set to $10^8$. Cycling at 2.5 V for 1000 cycles was performed prior to the retention measurements. In case of the Hf-rich samples, pre-cycling was extended to up to 4000 cycles in order to achieve similar 2P$_{\mathrm{S}}$ values. For the measurement of standard I–V and P–V characteristics, a 100 Hz triangular waveform with 2.3 V amplitude was used. C–V measurements were conducted with a small-amplitude (50 mV) sinusoidal signal of 1 kHz frequency, upon sweeping of DC bias between − 2.5 and 2.5 V (Fig. 5). FORC measurements were conducted with an maximum amplitude of ± 2.25 V^12^.

### Grazing incidence X-ray diffraction

Grazing incidence X-ray diffraction patterns were recorded with an incidence angle of 0.5$^\circ$ and 2$\theta$ ranging from 15$^\circ$ to 45$^\circ$.

## Supplementary Information

Supplementary Information.

## Data Availability

The data that support the findings of this study are included in the main text.

## References

[1] Böscke TS, Müller J, Bräuhaus D, Schröder U, Böttger U (2011). Ferroelectricity in hafnium oxide thin films. Appl. Phys. Lett..

[2] Müller, J., Yurchuk, E., Schlosser, T., Paul, J., Hoffmann, R., Müller, S., Martin, D., Slesazeck, S., Polakowski, P., Sundqvist, J., Czernohorsky, M., Seidel, K., Kucher, P., Boschke, R., Trentzsch, M., Gebauer, K., Schröder, U. & Mikolajick, T. Ferroelectricity in HfO2 enables nonvolatile data storage in 28 nm HKMG, in *IEEE Symposium on VLSI Technology*, VLSI (I. Staff 2 ed.) 25–26 (IEEE, 2012). 10.1109/VLSIT.2012.6242443.

[3] Toriumi, A., Xu, L., Mori, Y., Tian, X., Lomenzo, P. D., Mulaosmanovic, H., Materano, M., Mikolajick, T. & Schröder, U. Material perspectives of HfO2-based ferroelectric films for device applications, in * IEEE International Electron Devices Meeting*, IEDM ( IEEE, 2019) pp. 15.1.1–15.1.4 10.1109/IEDM19573.2019.8993464.

[4] Mart C, Kämpfe T, Zybell S, Weinreich W (2018). Layer thickness scaling and wake-up effect of pyroelectric response in Si-doped HfO2. Appl. Phys. Lett..

[5] Kirbach S, Lederer M, Eßlinger S, Mart C, Czernohorsky M, Weinreich W, Wallmersperger T (2021). Doping concentration dependent piezoelectric behavior of Si:HfO$$_2$$ thin-films. Appl. Phys. Lett..

[6] Hakim, F., Tharpe, T. & Tabrizian, R. Ferroelectric-on-Si Super-High-Frequency Fin Bulk Acoustic Resonators with Hf$$_{0.5}$$Zr$$_{0.5}$$O$$_2$$ Nano-Laminated Transducers, IEEE Microwave Wirel. Compon. Lett. 10.1109/LMWC.2021.3067509(2021).

[7] Müller J, Polakowski P, Müller S, Mikolajick T (2015). Ferroelectric hafnium oxide based materials and devices: Assessment of current status and future prospects. ECS J. Solid State Sci. Technol..

[8] Lehninger, D., Olivo, R., Ali, T., Lederer, M., Kämpfe, T., Mart, C., Biedermann, K., Kühnel, K., Roy, L., Kalkani, M. & Seidel, K. Back-end-of-line compatible low temperature furnace anneal for ferroelectric hafnium zirconium oxide formation. Phys. Status Solidi (a) ** 217**, 1900840 10.1002/pssa.201900840 (2020).

[9] Lehninger, D., Ali, T., Olivo, R., Lederer, M., Kämpfe, T., Mertens, K. & Seidel, K. Furnace annealed HfO2-films for the integration of ferroelectric functionalities into the BEoL, in *Joint conference of the IEEE international frequency control symposium and international symposium on applications of ferroelectrics (IFCS-ISAF)* 1–3 (IEEE, 2020). 10.1109/IFCS-ISAF41089.2020.9234879.

[10] Lederer M, Olivo R, Lehninger D, Abdulazhanov S, Kämpfe T, Kirbach S, Mart C, Seidel K, Eng LM (2021). On the origin of wake-up and antiferroelectric—like behavior in ferroelectric hafnium oxid. Phys. Status Solidi (RRL).

[11] Pesic M, Schroeder U, Slesazeck S, Mikolajick T (2018). Comparative study of reliability of ferroelectric and anti-ferroelectric memories. IEEE Trans. Dev. Mater. Reliab..

[12] Schenk T, Yurchuk E, Müller S, Schröder U, Starschich S, Böttger U, Mikolajick T (2014). About the deformation of ferroelectric hystereses. Appl. Phys. Rev..

[13] Reyes-Lillo SE, Garrity KF, Rabe KM (2014). Antiferroelectricity in thin-film ZrO$$_2$$ from first principles. Phys. Rev. B.

[14] Hoffmann M, Schröder U, Künneth C, Kersch A, Starschich S, Böttger U, Mikolajick T (2015). Ferroelectric phase transitions in nanoscale HfO2 films enable giant pyroelectric energy conversion and highly efficient supercapacitors. Nano Energy.

[15] Nishimura T, Xu L, Shibayama S, Yajima T, Migita S, Toriumi A (2016). Ferroelectricity of nondoped thin HfO2 films in TiN/HfO2/TiN stacks. Jpn. J. Appl. Phys..

[16] Mimura T, Shimizu T, Katsuya Y, Sakata O, Funakubo H (2020). Thickness- and orientation- dependences of Curie temperature in ferroelectric epitaxial Y doped HfO2 films. Jpn. J. Appl. Phys..

[17] Shimizu, T., Tashiro, Y., Mimura, T., Kiguchi, T., Shiraishi, T., Konnno, T. J., Sakata, O. & Funakubo, H. Electric-field-induced ferroelectricity in 5%Y-doped Hf0.5Zr0.5O2: Transformation from the paraelectric tetragonal phase to the ferroelectric orthorhombic phase. Phys. Status Solidi (RRL). 10.1002/pssr.202000589 (2021).

[18] Tashiro, Y., Shimizu, T., Mimura, T. & Funakubo, H. Comprehensive study on the kinetic formation of the orthorhombic ferroelectric phase in epitaxial Y-doped ferroelectric HfO2 thin films. *ACS Appl. Electron. Mater.*10.1021/acsaelm.1c00342 (2021).

[19] Dragoman M, Modreanu M, Povey IM, Iordanescu S, Aldrigo M, Romanitan C, Vasilache D, Dinescu A, Dragoman D (2017). Very large phase shift of microwave signals in a 6 nm Hf$$_{{\rm x}}$$Zr$$_{{\rm 1-x}}$$O$$_{2}$$ Ferroelectric at $$\pm$$3 V. Nanotechnology.

[20] Abdulazhanov, S., Le, Q. H., Huynh, D. K., Wang, D., Gerlach, G. & Kämpfe, T. A mmWave Phase Shifter Based on Ferroelectric Hafnium Zirconium Oxide Varactors, in * 2019 IEEE MTT-S international microwave workshop series on advanced materials and processes for RF and THz applications (IMWS-AMP)* 175–177 (IEEE, 2019). 10.1109/IMWS-AMP.2019.8880144

[21] Abdulazhanov, S., Le, Q. H., Huynh, D. K., Wang, D., Gerlach, G., Kämpfe, T. A Tunable mmWave band-pass filter based on ferroelectric hafnium zirconium oxide varactors, in * 2019 IEEE MTT-S international microwave workshop series on advanced materials and processes for RF and THz applications (IMWS-AMP)* 46–48 (IEEE, 2019). 10.1109/IMWS-AMP.2019.8880098.

