# Supplementary material for: Electric field-induced crystallization of ferroelectric hafnium zirconium oxide
Source: Sci Rep. 2021 Nov 15;11:22266. doi: 10.1038/s41598-021-01724-2 (PMC8594776; doi:10.1038/s41598-021-01724-2)
Supplement: Supplementary file 1 — Supplementary Information. [file 41598_2021_1724_MOESM1_ESM.pdf]

Electric field-induced crystallization of ferroelectric hafnium zirconium oxide - supplementary information

Maximilian Lederer[1,*], Sukhrob Abdulazhanov[1], Ricardo Olivo[1], David Lehninger[1], Thomas Kämpfe[1], Konrad Seidel[1], and Lukas M. Eng[2,3]
[1]*Fraunhofer IPMS, Center Nanoelectronic Technologies, 01109 Dresden, Germany*
[2]*Institut für Angewandte Physik, Technische Universität Dresden, 01062 Dresden, Germany*
[3]*Center of Excellence - Complexity and Topology in Quantum Matter (ct.qmat), Technische Universität Dresden, 01062 Dresden, Germany.*
[*]*e-mail: maximilian.lederer@ipms.fraunhofer.de*

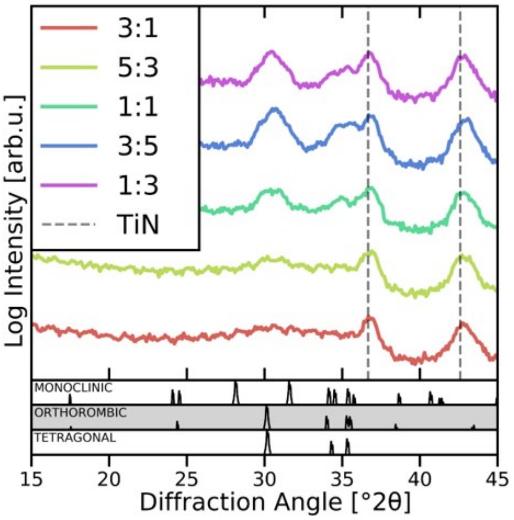

Figure S1. Grazing-incident X-ray diffraction patterns of HZO films with different Hf:Zr cycling ratio. Transition from amorphous to (semi-)crystalline films is observed with increasing Zr content.

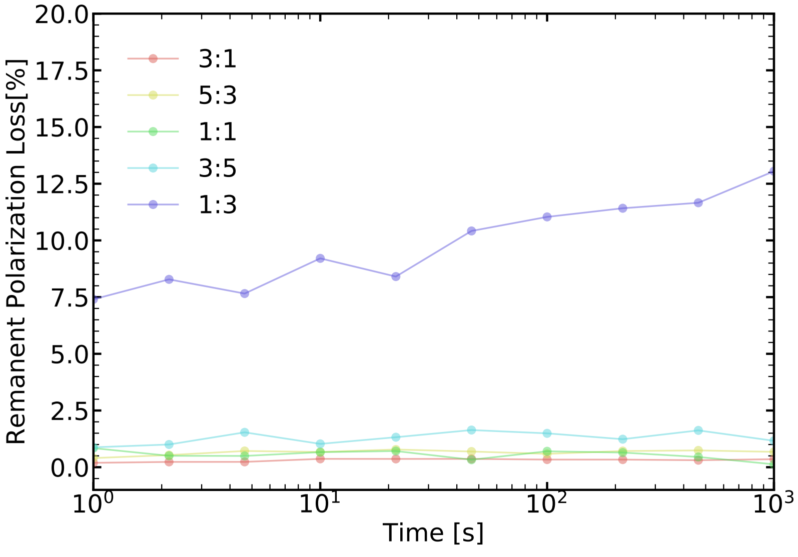

Figure S2. Retention of the remanent polarization of the the HZO films with different ALD cycling ration (Hf:Zr). No significant losses are observed, except for the strongly AFE-like and Zr-rich sample, which is an artifact of resulting from the imprint effect in AFE-like layers.

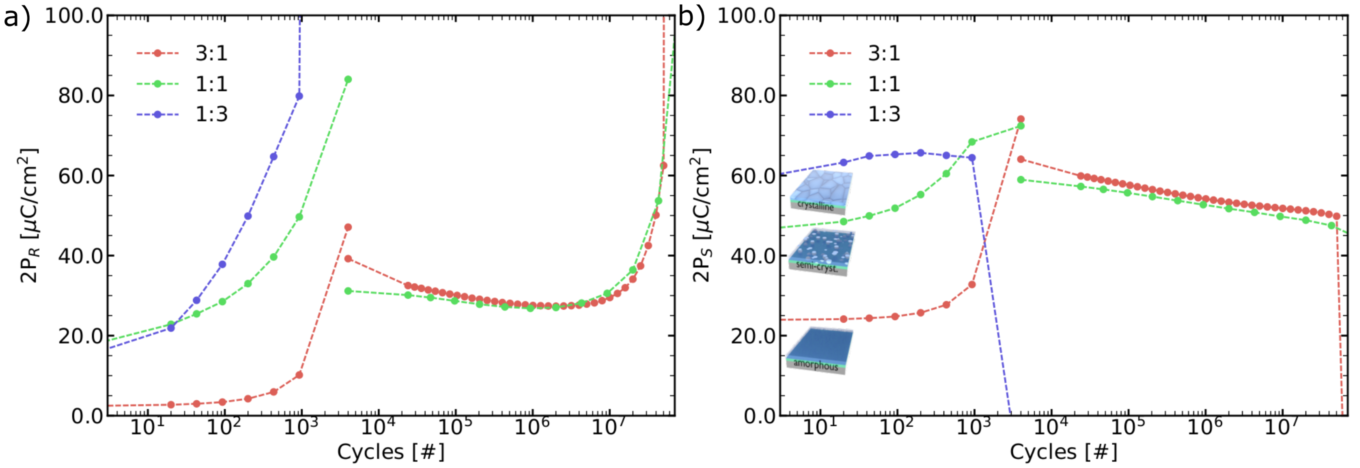

Figure S3. Endurance characteristics for P_R (a) and P_S (b). After initial wake-up cycling at 2.3 V and 20 Hz, endurance was measured with field cycling at 1.9 V. Soft breakdown is observed for the initially semi-crystalline and amorphous samples. The Zr-rich sample (Hf:Zr 1:3) already exhibits hard break down during wake-up cycling.

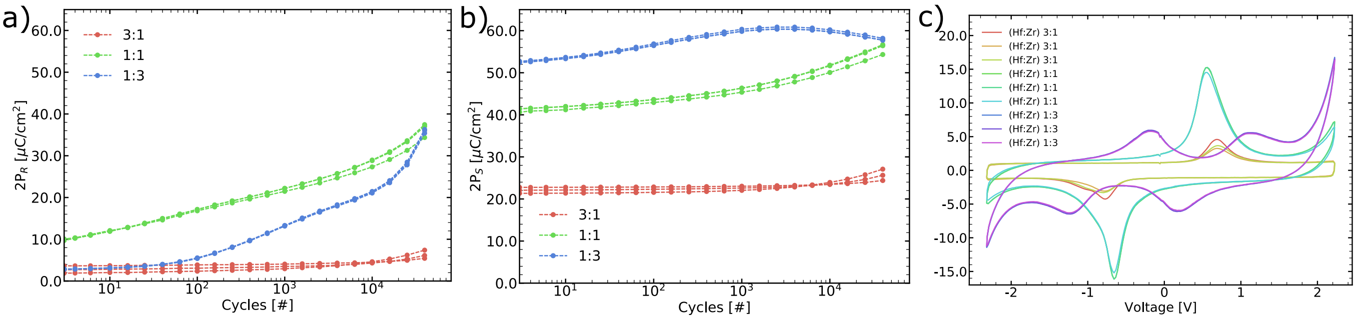

Figure S4. Endurance characteristics for P_R (a) and P_S (b) starting from pristine state at 2.3 V and 1 kHz. Very similar behavior is observed for different devices. Analogue to the wake-up measurement with low frequency (Fig. S3), earlier breakdown is observable for the Zr-rich sample. This is strongly driven by increased leakage current, which can be clearly seen in the displacement current (c) after $3.98 \cdot 10^4$ cycles.
